# Dehydroepiandrosterone plus climen supplementation shows better effects than dehydroepiandrosterone alone on infertility patients with diminished ovarian reserve of low-FSH level undergoing in-vitro fertilization cycles: a randomized controlled trial

**DOI:** 10.1186/s12958-016-0139-z

**Published:** 2016-02-16

**Authors:** Huanhuan Zhang, Yaping Chu, Ping Zhou, Xiaojin He, Qianhua Xu, Zhiguo Zhang, Yunxia Cao, Zhaolian Wei

**Affiliations:** Department of Reproductive Endocrinology, The Reproductive Medicine Center, The Affiliated Hospital of Anhui Medical University, Hefei, Anhui, People’s Republic of China; Hangzhou Women’s Health Hospital, Zhejiang, China

**Keywords:** Dehydroepiandrosterone, Oestradiol valerate and cyproterone acetate drug combination, Diminished ovarian reserve, In-vitro fertilization

## Abstract

**Background:**

This study aimed to assess the effect of dehydroepiandrosterone (DHEA) plus climen (estradiol valerate and cyproterone acetate drug combination) on infertility patients with diminished ovarian reserve (DOR) and to determine if the combination of DHEA plus climen is superior to DHEA alone in improving ovarian response.

**Methods:**

A total of 124 women were randomized into the DHEA group (*n* = 64) and the DHEA plus climen group (*n* = 60) for 12 weeks before being subjected to in-vitro fertilization (IVF) cycles. To investigate if there is a FSH-related difference on the effect of the addition of climen, the DHEA group and the DHEA plus climen group were further divided into four subgroups according to a basal FSH level cut-off of 10 mIU/ml. We performed a comparison of Day 3 blood samples before and after treatment and IVF outcome parameters, including AMH, FSH, E2, AFC, oocytes retrieved, MII oocyte numbers, embryo numbers and accumulated embryo scores.

**Results:**

After 12 weeks of pretreatment, the DHEA plus climen group demonstrated a significantly higher level of AMH (*P* = 0.001) and a significantly lower level of FSH (*P* = 0.001) compared with the DHEA group. When the two groups were divided into four subgroups based on the FSH cut-off of 10 mIU/mL, a significant increase of AMH (*P* = 0.034) was found in the high-FSH DHEA plus climen group, whereas there was no significant difference in the high-FSH DHEA group (*P* = 0.322). A significantly higher accumulated score of embryos was observed in the low-FSH DHEA plus climen group compared with the low-FSH DHEA group (*P* = 0.034).

**Conclusions:**

These observations suggest that patients with DOR of a low-FSH level might benefit more from DHEA plus climen supplementation than from DHEA supplementation alone.

## Background

Dehydroepiandrosterone (DHEA), the body’s most abundant circulating steroid substance, is produced by primarily by the adrenal zona reticularis and ovarian theca cells and functions as a precursor of testosterone and oestradiol in the periphery [1]. The essential role of androgens is to facilitate normal follicle development in the mouse. Androgens appear most engaged at the pre-antral and antral stages, primarily affect granulosa cells, and exert effects via androgen receptors (ARs), with ligand-activated AR modulating follicle stimulating hormone (FSH) activity in granulosa cells. Therefore, androgens serve as important modulators of granulosa cell differentiation and follicle maturation [2].

However, the effects of DHEA supplementation remain controversial in humans because of a lack of large, well designed, optimal quality randomized controlled trials (RCTs). Previous studies [3–5] reported that the addition of DHEA could improve oocyte and embryo yields, oocyte and embryo quality, pregnancy rates and reduce miscarriage risk in patients with diminished ovarian reserve (DOR). However, many of the early studies of DHEA supplementation were not well accepted and/or were based on observational data, and the results of those studies were not free from bias. A recent RCT [6] revealed that no statistically significant differences were found in the ovarian response markers (AFC, AMH, or FSH) or IVF outcomes between these two groups, although the serum DHEA-S, free androgen index, and follicular DHEA-S levels were significantly increased upon DHEA supplementation. However, this study was limited by its small sample size of 32 participants.

Given that experiments in rodents strongly support an essential androgen function and there is a controversial effect in humans, the current study continues to investigate the use of DHEA and to determine the optimum treatment regimen for patients with DOR.

Patients with DOR have a progressive decline in ovarian oocyte quality and quantity, with an elevated basal FSH level and a lower antral follicle count than patients without DOR. Research has indicated that pretreatment with oestrogen might inhibit concentrations of circulating FSH, restricting the depletion of follicles [7]. DHEA, as described above, could up-regulate FSH receptors, which might make remnant follicles more sensitive to FSH administration. Climen (estradiol valerate and cyproterone acetate drug combination), which mimics the function of oestrogen, could maintain the menstrual cycle and does not inhibit ovulation. Therefore, we question if DHEA/climen synergism could further augment the beneficial effects of DHEA on ovarian reserve.

To evaluate this hypothesis, predictors of ovarian reserve including serum AMH, FSH, and E_2_ of all patients were measured pre- and post-treatment in each group. IVF cycle outcomes, including the number of oocytes retrieved, MII oocytes, embryos and accumulated scores of embryos, were compared between the two groups.

## Methods

### Patients and design

This prospective study was conducted from July 2014 to May 2015 at the Assisted Reproductive Center, The Affiliated Hospital of Anhui Medical University. The study was approved by the Clinical Research Ethics Committee of The First Affiliated Hospital of Anhui Medical University (PJ-20140515) and Chinese Clinical Trial Register (ChiCTR-TRC-14005030). Before the trial, the possible effects of pretreatment and the trial were fully explained to each participant and written consents were obtained.

We included infertility patients with diminished ovarian reserve undergoing in vitro fertilization cycles. The inclusion criteria for defining DOR in this study were defined as follows: 1) an elevated day 3 (on the third day of menstrual cycle) FSH level ≥10 mIU/mL or FSH/LH >3; 2) an antral follicle count less than five; 3) a previous poor ovarian response of retrieval of fewer than five oocytes or cycle cancellation due to poor response to ovarian stimulation. A diagnosis of DOR was reached if the patient fulfilled any one of the above three criteria. Patients were excluded if they had any of the following: 1) a history of ovarian cystectomy or oophorectomy; 2) a diagnosis of endometriosis; 3) a history of DHEA supplementation or hormonal replacement therapy; 4) abnormal thyroid, liver or kidney function. Only Chinese women were included in the study because DHEA acts mainly via conversion to testosterone, and the rate of this conversion could be quite different in different populations.

Participants were randomized in a 1:1 ratio according to a computer-generated randomization sequence by a study nurse and were allocated using sealed, opaque, sequentially numbered envelopes. Neither the study nurse nor the participants would not know which group they were in until the envelopes were opened by the studiers. Patients in the DHEA group received a supplementation of DHEA 25 mg three times per day. Patients in the DHEA plus group would be supplemented with climen (Climen, Bayer, German) 1 mg once per day.

### Sample collection and determination

On the third day of the menstrual cycle (day 3), blood samples were collected for the detection of serum AMH, FSH, and E2 before the supplementation was started in the groups. After 12 weeks of therapy prior to ovarian stimulation, day 3 blood samples were collected again to detect serum AMH, FSH, and E2, and the results were compared with the samples collected prior to therapy. The serum samples were centrifuged at 2000 r/min for 15 min and stored at −80 °C until they were tested.

The concentration of AMH was determined by an enzyme-linked immunosorbent assay (ELISA) [AMH: AMH Gen II ELISA, ASNL, USA]. FSH and E_2_ levels in serum were determined by a radioimmunoassay kit (Beckman Coulter, Brea, USA).

### Ovarian stimulation and embryo grade

After 12 weeks of DHEA or DHEA plus climen therapy, patients with DOR were given 225 IU human menopausal gonadotropin (HMG, Lizhu, China) from day 3 of the cycle with 100 mg clomiphene citrate (CC, Lizhu, China) once a day. Serial trans-vaginal scans were performed using a 6.0 MHz vaginal probe (Toshiba SSA-550A; Japan) to monitor the growth of follicles in both ovaries. When at least one mature follicle with a mean diameter ≥18 mm was observed on ultrasound, 10,000 IU of human chorionic gonadotropin (HCG, Lizhu, China) was administered, followed by trans-vaginal oocyte retrieval 36 h later. The participants would then undergo an IVF or ICSI cycle depending on the quality of their partner’s sperm.

Based on the percent fragmentation and cell counts, embryos were graded from one to four on the third day of incubation. Up to three of the best-quality embryos were transferred on day 3. The accumulated score of embryos was calculated by summing the score of each embryo produced by each patient on the third day of development. For example, a patient with two grade 4 embryos and one grade 3 embryo would be assigned a score of 11. Patients with cycle cancellation would be assigned a score of 0.

### Clinical pregnancy

Fourteen days after the embryo transfer, a quantitative pregnancy test (serum β-hCG) was taken. If positive, a trans-vaginal ultrasound was performed 20 days later. Clinical pregnancy was confirmed if a foetal heartbeat was observed.

### Statistical analyses

SPSS 17.0 was utilized for the data analysis. The data were presented as the mean ± standard deviation (SD) or as percentages. Quantitative variables were analysed with Student’s t-test or the Mann-Whitney-U test, followed by the one-sample Kolmogorov-Smirnov test. Quality variables were analysed with the chi-square test. A two-sided *P* < 0.05 was considered statistically significant.

## Results

**Fig. 1:**
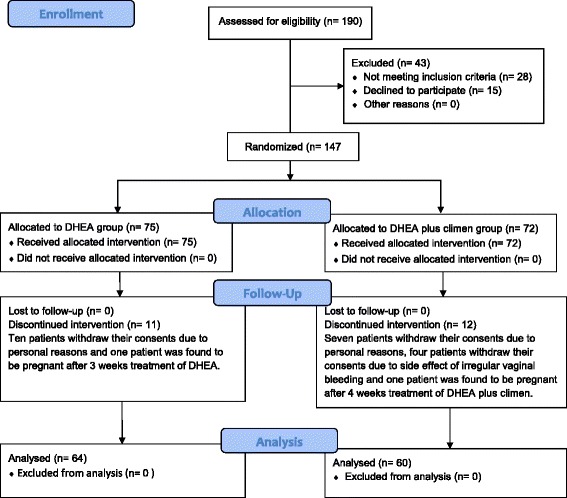
Flow diagram

This study was conducted from July 2014 to May 2015. During this period, a total of 147 patients fulfilled the criteria of DOR and consented to participate this research. After excluding discontinuers, 64 patients were exposed to DHEA treatment, and 60 patients were exposed to DHEA plus climen treatment for 12 weeks (See Fig. 1). All women in this study underwent only one IVF or ICSI cycle according to the quality of their spouse’s sperm.

As summarized in Table 1, the two groups were homogeneous in terms of age, BMI, type of infertility and infertility durations (*P* = 0.929, 0.644, 0.961 and 0.761, respectively). No significant difference was found regarding the basal AMH, FSH, E2, and antral follicle count (AFC), which represent ovarian reserve in the two groups (*P* = 0.466, 0.343, 0.719 and 0.059, respectively).

**Table 1:** Comparison of baseline characters between the two groups

	The DHEA group *n* = 64	The DHEA plus climen group *n* = 60	*P*-value
Age (years)	37.06 ± 5.06	37.15 ± 5.80	0.929
BMI (kg/m^2^)	22.18 ± 2.73	22.39 ± 2.48	0.644
Primary infertility	42.19 %	42.67 %	0.961
Secondary infertility	57.81 %	57.33 %	
Infertility duration (years)	6.39 ± 4.10	6.65 ± 5.32	0.761
AMH (ng/ml)	0.98 ± 0.72	0.90 ± 0.66	0.466
FSH (IU/L)	12.93 ± 7.02	11.86 ± 5.29	0.343
E2 (pmol/L)	181.53 ± 137.26	160.65 ± 116.90	0.719
AFC	3.25 ± 2.07	3.47 ± 1.95	0.059

Data are presented as mean ± SD (standard deviation) or percentage as appropriate

*AFC* the count of antral follicle

*P* < 0.05 was considered as statistically significant

As shown in Table 2, after 12 weeks of treatment with DHEA plus climen, the mean serum AMH level was significantly higher (0.90 ± 0.66 vs. 1.14 ± 0.79, *P* =0.001), and the FSH level was significantly lower (11.86 ± 5.29 vs. 9.08 ± 5.51, *P* = 0.001) compared with pre-treatment levels. However, a significantly higher level of E_2_ (160.65 ± 116.90 vs. 278.50 ± 135.80, *P* = 0.000) was observed in this group compared with the pre-treatment level.

**Table 2:** Comparison of the ovarian reserve markers pre- and post-treatment in each group

	The DHEA group		*P*-value	The DHEA plus climen group		*P*-value
	Pre-treament	Post-treatment		Pre-treament	Post-treatment	
AMH (ng/ml)	0.98 ± 0.72	1.24 ± 1.07	0.015	0.90 ± 0.66	1.14 ± 0.79	0.001
FSH(IU/L)	12.93 ± 7.02	10.03 ± 5.48	0.003	11.86 ± 5.29	9.08 ± 5.51	0.001
E2 (pmol/L)	181.53 ± 137.26	113.96 ± 70.00	0.001	160.65 ± 116.90	278.50 ± 135.80	0.000
High-FSH subgroups						
AMH (ng/ml)	0.88 ± 0.65	0.99 ± 1.06	0.322	0.68 ± 0.58	0.89 ± 0.75	0.034
FSH (IU/L)	17.35 ± 6.34	11.84 ± 6.45	0.001	15.40 ± 4.00	10.56 ± 6.54	0.001
E2 (pmol/L)	155.70 ± 93.43	122.32 ± 79.44	0.070	120.35 ± 81.91	276.30 ± 139.76	0.000
Low-FSH subgroups						
AMH (ng/ml)	1.11 ± 0.78	1.56 ± 1.01	0.002	1.18 ± 0.67	1.45 ± 0.74	0.004
FSH (IU/L)	7.24 ± 1.82	7.69 ± 2.49	0.445	7.13 ± 2.11	7.14 ± 2.87	0.982
E2 (pmol/L)	214.75 ± 174.96	103.21 ± 55.14	0.003	213.37 ± 135.26	281.53 ± 133.33	0.035

Data are presented as mean ± SD (standard deviation) or percentage as appropriate. Subgroups were divided based on a basal FSH level cut-off of 10 mlU/ml

*P* <0.05 was considered as statistically significant

A comparison of ovarian reserve markers was made in the DHEA group pre- and post-treatment. A significant increase in the AMH (0.98 ± 0.72 vs. 1.24 ± 1.07, *P* = 0.015) and a significant decrease in the FSH (12.93 ± 7.02 vs. 10.03 ± 5.48, *P* = 0.003) were observed in patients treated with DHEA. The E_2_ level (181.53 ± 137.26 vs. 113.96 ± 70.00, *P* = 0.001) was significantly lower in this group after treatment with DHEA (Table 2).

We monitored the concentration of DHEA-S and testosterone in this trial. DHEA-S and testosterone levels were significantly higher after treatment in the DHEA + climen group (1.14 ± 0.68 μg/mL vs. 4.73 ± 3.04 μg/mL, *P* =0.00; 0.59 ± 0.51 nmol/L vs. 1.98 ± 1.14 nmol/L, *P* =0.00) and in the DHEA group (1.15 ± 0.64 μg/mL vs. 5.35 ± 3.28 μg/mL, *P* =0.00; 0.63 ± 0.40 nmol/L vs. 2.05 ± 1.12 nmol/L, *P* =0.00).

To investigate if there is a different FSH-related effect, the DHEA group and DHEA plus climen group were further divided into four subgroups according to a basal FSH level cut-off of 10 mIU/mL. Table 2 shows a significantly higher level of AMH (0.68 ± 0.58 vs. 0.89 ± 0.75, *P* = 0.034) in the high-FSH DHEA plus climen subgroup post-treatment (*n* = 34), whereas there was no significant difference in the high-FSH DHEA subgroup pre- and post-treatment (0.88 ± 0.65 vs. 0.99 ± 1.06, *P* = 0.322; *n* = 36). Both the DHEA group and the DHEA plus climen group showed a significant decrease in FSH post-treatment.

A significantly higher level of AMH was observed in the low-FSH DHEA plus climen subgroup (1.18 ± 0.67 vs. 1.45 ± 0.74, *P* = 0.004; *n* = 26) and in patients in the low-FSH DHEA subgroup (1.11 ± 0.78 vs. 1.56 ± 1.01, *P* = 0.002, *n* = 28). However, neither the DHEA group nor the DHEA plus climen group showed significant changes in the level of FSH after 12 weeks of treatment (Table 2).

**Fig. 2:**
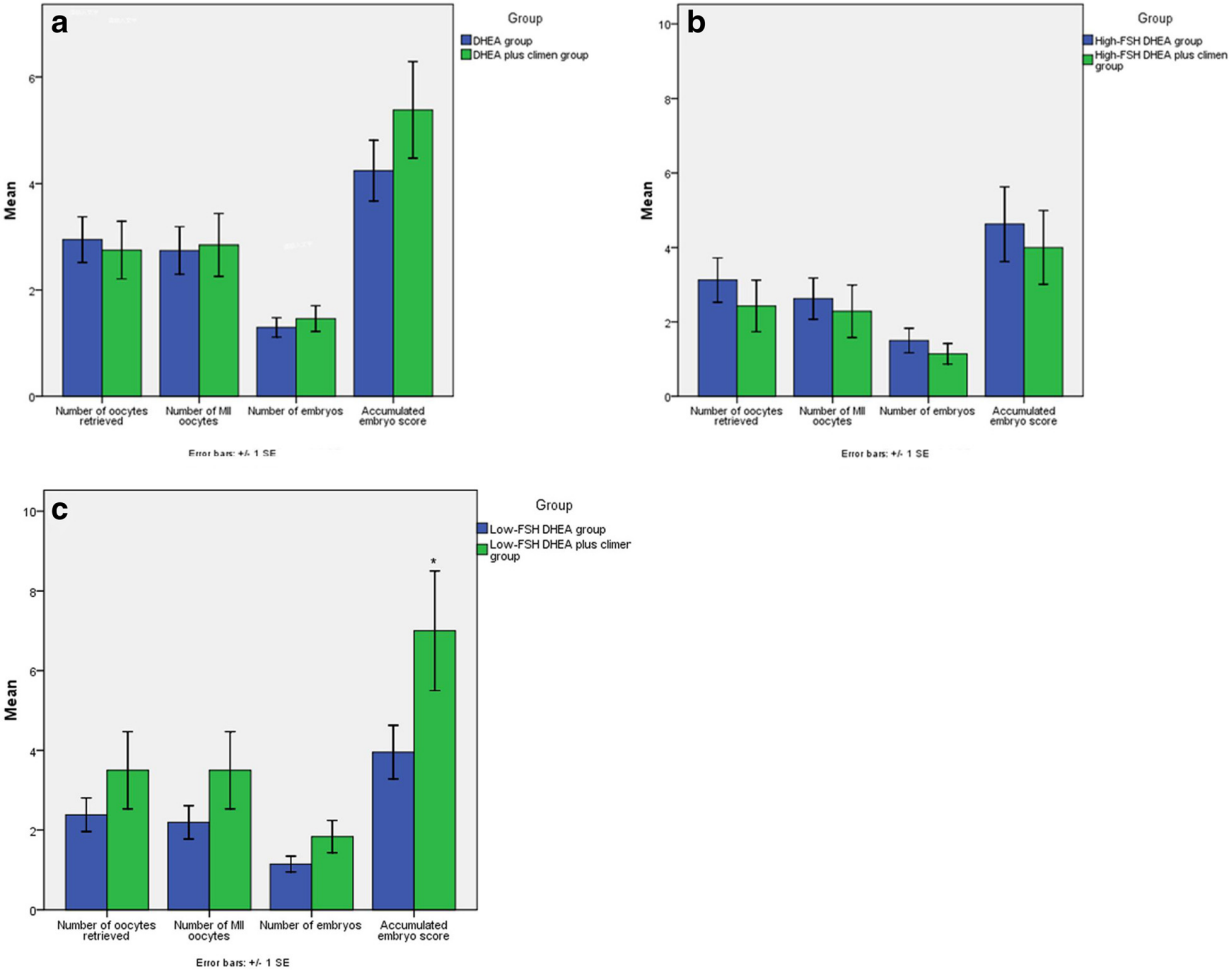
**a** Comparisons of IVF cycle outcomes between the DHEA plus climen group and the DHEA group. **b** Comparisons of IVF cycle outcomes between the two high-FSH sub-groups. **c** Comparisons of IVF cycle outcomes between the two low-FSH sub-groups. *P <0.05

IVF cycle outcomes were compared as shown in Fig. 2. Comparable effects including the number of oocytes retrieved, MII oocytes, embryos and accumulated score of embryos were observed in the DHEA plus climen group and the DHEA group (*P* = 0.862, 0.355, 0.354 and 0.196, respectively; Fig. 2a). There were no significant differences in the IVF outcomes between the two High-FSH subgroups (Fig. 2b). However, a significantly higher accumulated embryo score was observed in the low-FSH DHEA plus climen group compared with the low-FSH DHEA group (*P* = 0.034, Fig. 2c).

A total of 93 embryos (52 in the Low-FSH subgroup, 41 in the High-FSH subgroup) were achieved in the DHEA + climen group, and 85 embryos were achieved in the DHEA group (46 in the Low-FSH subgroup, 39 in the High-FSH subgroup). The implantation rate of the low-FSH DHEA + climen group was 17.31 % higher than the Low-FSH DHEA group (13.04 %), although the difference was not statistically significant (*P* = 0.196). No significant differences were found in the implantation rates between the DHEA + climen group and the DHEA group (16.13 vs. 12.94 %, *P* = 0.242) or between the two High-FSH sub-groups (14.63 vs. 12.82 %, *P* = 0.446).

Fourteen clinical pregnancies (8 in the Low-FSH subgroup, 6 in the High-FSH subgroup) were achieved in the DHEA plus climen group after the IVF cycle, with a pregnancy rate of 23.33 %. One pregnancy in the Low-FSH subgroup was a twin pregnancy. In the DHEA group, 11 patients (6 in the Low-FSH subgroup, 5 in the High-FSH subgroup) conceived, with a pregnancy rate of 17.19 %. There were no significant differences in the pregnancy rates between the DHEA plus climen group and the DHEA group (*P* = 0.616), between the two low-FSH subgroups (*P* = 0.946) or between the two high-FSH subgroups (*P* = 0.743).

In this study, we used CC + HMG, and the results are limited to this treatment.

## Discussion

Because patients with DOR are faced with limited remaining reproductive life spans and live under great pressure, treatments for these women need improvement and increased evidence.

The rationale for this study was based on the hypothesis that a chronically high FSH level in patients with DOR down-regulates granulosa-cell FSH receptors, causing the remaining follicles to become refractory to exogenous stimulation. A study suggested that oestrogen might increase the FSH receptors on granulosa-cells and enhance the binding of FSH to its receptors [8]. Pretreatment with oestrogens in patients affected by premature ovarian failure down-regulated the level of FSH and improved the rate of ovulation induction [9]. Moreover, researchers determined that pregnancy could be achieved in patients with premature ovarian failure after treatment with oestrogen plus progesterone [10]. Herein, we substituted climen for the component oestrogen to maintain the menstrual cycle.

Previous studies [11, 12] identified the potential role of DHEA. Androgens play an essential role in normal follicle maturation. Androgens primarily affect granulosa cells and exert effects with ligand-activated ARs [11]. According to the two cell/two gonadotropin theory, it has been postulated that DHEA could improve steroidogenesis within the ovary because DHEA is a crucial precursor steroid to human sex steroid synthesis [13]. DHEA plays an important role in the form of testosterone and E2 in the periphery. Testosterone and its metabolite 5α-dehydrotestosterone (DHT) are the best agonists for the ARs compared with other androgens [14]. Up-regulated ARs further modulate FSH activities in granulosa cells and modulates follicle maturation [2]. Granulosa cells respond to FSH and induce changes in androgen metabolism. There appears to be a feedback loop between androgens and FSH. Therefore, DHEA serves as an important modulator of follicle maturation.

In animal models, follicle recruitment is under the synergism of FSH/androgen [15]. In humans, researchers have observed that repeated short (<120 days) interval exposure to DHEA resulted in increased oocyte yield across three consecutive IVF cycles. This observation suggested that patients with DOR might take advantage of a possible DHEA/FSH synergism [16].

To evaluate the effect of DHEA plus climen on ovarian reserve, serum AMH, FSH, and E_2_ were detected in the present study. The serum AMH and FSH levels and the number of antral follicles are the three most frequently used predictors for ovarian reserve prior to IVF cycles in clinical practice [17]. Furthermore, AMH is the single best marker predicting ovarian response to gonadotropins and defines low versus good live-birth chances in women with severely diminished ovarian reserve independent of age [18].

There was a significantly higher level of AMH and a significantly lower level of FSH in the patients treated with DHEA plus climen. Animal experiments demonstrated that the expression of AR mRNA was positively correlated with the expression of FSH receptor mRNA. Moreover, the expression of AR mRNA showed a positive association with the AMH receptor. Therefore, the increase of AMH and the decrease of FSH in our study are understandable results [19].

Similarly, comparable effects including a significant increase in AMH and a significant decrease in FSH were observed in the DHEA group. This result is consistent with previous studies. Yilmaz reported a significant difference in all of the parameters after at least 6 weeks of DHEA treatment [20]. Neeta Singh obtained a similar result after studying 30 patients with a history of poor response. These women were treated with DHEA for 4 months longer than previous study. A significant increase in serum AMH levels and a significant decrease in Day 2 FSH levels were observed in all age groups (35, 36–38 and >38 years) [21]. However, no statistically significant change was detected in the antral follicle count.

A recent RCT observed no significant differences in serum FSH and AMH levels between the DHEA group and the placebo group throughout the study period, although this result is not in agreement with previous studies [6]. However, the level of follicular DHEA-S was significantly higher in the DHEA group. Follicular AMH was also higher in the DHEA group, although the increase did not reach statistical significance.

A significant increase in E_2_ was observed in the DHEA plus climen group, whereas the level was significantly lower in patients after treatment with DHEA. This result might be interpreted as the exogenous complement of climen because E_2_ is the combination of oestradiol valerate and cyproterone acetate.

Both subgroups with low FSH level demonstrated a significant increase in AMH. However, neither group showed a significant change in FSH. These results are expected because the patients within these two subgroups had normal FSH values lower than 10 mIU/ml and were diagnosed with DOR because of a history of a poor response or an antral follicle count of less than five. The increased AMH indicated the effect of DHEA plus climen group on patients with normal FSH.

Both sub-groups with high FSH level showed a significant decrease in the level of FSH. However, only the patients in the DHEA plus climen group had a significant change in the level of AMH. Moreover, a significantly higher level of accumulated embryo score was observed in the low-FSH DHEA plus climen group compared with the low-FSH DHEA group. Despite the small size of our study group, the differences between the groups are marked.

## Conclusions

DHEA plus climen supplementation prior to in-vitro fertilization (IVF) cycles might have better effects than DHEA alone for infertility patients with diminished ovarian reserve of low-FSH level.

## References

[1] Burger HG (2002). Androgen production in women. Fertil Steril.

[2] Lenie S, Smitz J (2009). Functional AR signaling is evident in an in vitro mouse follicle culture bioassay that encompasses most stages of folliculogenesis. Biol Reprod.

[3] Barad D, Gleicher N (2006). Effect of dehydroepinadrosterone on oocytes and embryo yields, embryo grade and cell number in IVF. Hum Reprod.

[4] Barad D, Brill H, Gleicher N (2007). Update on the use of dehydroepiandrosterone supplementation among women with diminished ovarian function. J Assist Reprod Genet.

[5] Gleicher N, Ryan E, Weghofer A, Blanco-Mejia S, Barad DH (2009). Miscarriage rates after dehydroepiandrosterone (DHEA) supplementation in women with diminished ovarian reserve: a case control study. Reprod Biol Endocrinol.

[6] Li RH, Lee VC, Ho PC, Ng EH (2014). A randomized, controlled, pilot trial on the effect of dehydroepiandrosterone on ovarian response markers, ovarianresponse, and in vitro fertilization outcomes in poor responders. Fertil Steril.

[7] Check JH, Nowroozi K, Chase JS, Nazari A, Shapse D, Vaze M (1990). Ovulation induction and pregnancies in 100 consecutive women with hypergonadotropic amenorrhea. Fertil Steril.

[8] Van Kasteren YM, Hoek A, Schoemaker J (1995). Ovulation induction in premature ovarian failure: a placebo-controlled randomized trial combining pituitary suppression with gonadotropin stimulation. Fertil Steril.

[9] Tartagni M, Cicinelli E, De Pergola G, De Salvia MA, Lavopa C, Loverro G (2007). Effects of pretreatment with estrogens on ovarian stimulation with gonadotropins in women with premature ovarian failure: a randomized, placebo-controlled trial. Fertil Steril.

[10] Zargar AH, Salahuddin M, Wani AI, Bashir MI, Masoodi SR, Laway BA (2000). Pregnancy in premature ovarian failure: a possible role of estrogen plus progesterone treatment. J Assoc Physicians India.

[11] Gleicher N, Weghofer A, Barad DH (2011). The role of androgens in follicle maturation and ovulation induction: friend or foe of infertility treatment?. Reprod Biol Endocrinol.

[12] Gleicher N, Weghofer A, Lee IH, Barad DH (2011). Association of FMR1 genotypes with in vitro fertilization (IVF) outcomes based on ethnicity/race. PLoS One.

[13] Gleicher N, Weghofer A, Barad DH (2010). Improvement in diminished ovarian reserve after dehydroepiandrosterone supplementation. Reprod Biomed Online.

[14] Lutz LB, Jamnongjit M, Yang W-H, Jahani D, Gill A, Hammes SR (2003). Selective modulation of genomic and nongenomic androgen responses by androgen receptor ligands. Mol Endocrinol.

[15] Sen A, Hammes SR (2010). Granulosa cell-specific androgen receptors are critical regulators of ovarian development and function. Mol Endocrinol.

[16] Barad DH, Kushnir VA, Lee HJ, Lazzaroni E, Gleicher N (2014). Effect of inter-cycle interval on oocyte production in humans in the presence of the weak androgen DHEA and follicle stimulating hormone: a case-control study. Reprod Biol Endocrinol.

[17] Ferraretti AP, La Marca A, Fauser BC, Tarlatzis B, Nargund G, Gianaroli L (2011). ESHRE consensus on the definition of ‘poor response’to ovarian stimulation for in vitro fertilization: the Bologna criteria. Hum Reprod.

[18] Gleicher N, Weghofer A, Barad DH (2010). Anti-Müllerian hormone (AMH) defines, independent of age, low versus good live birth chances in women with severely ovarian reserve. Fertil Steril.

[19] Nielsen ME, Rasmussen IA, Kristensen SG, Christensen ST, Møllgård K, Wreford Andersen E (2011). In human granulosa cells from small antral follicles, androgen receptor mRNA and androgen levels in follicular fluid correlate with FSH receptor mRNA. Mol Hum Reprod.

[20] Yilmaz N, Uygur D, Inal H, Gorkem U, Cicek N, Mollamahmutoglu L (2013). Dehydroepiandrosterone supplementation improves predictive markers for diminished ovarian reserve: serum AMH, inhibin B and antral follicle count. Eur J Obstet Gynecol Reprod Biol.

[21] Singh N, Zangmo R, Kumar S, Roy KK, Sharma JB, Malhotra N (2013). A prospective study on role of dehydroepiandrosterone (DHEA) on improving the ovarian reserve markers in infertile patients with poor ovarian reserve. Gynecol Endocrinol.

